# The Clinical and Imaging Characteristics Associated With Neurological Sequelae of Pediatric Patients With Acute Necrotizing Encephalopathy

**DOI:** 10.3389/fped.2021.655074

**Published:** 2021-05-11

**Authors:** Hong-Min Zhu, Si-Min Zhang, Cong Yao, Meng-Qing Luo, Hui-Jing Ma, Tao Lei, Chun-Hui Yuan, Ge-Fei Wu, Jia-Sheng Hu, Chun-Quan Cai, Zhi-Sheng Liu

**Affiliations:** ^1^Pediatric Clinical College of Tianjin Medical University, Tianjin, China; ^2^Department of Neurology, Wuhan Children's Hospital, Tongji Medical College, Huazhong University of Science and Technology, Wuhan, China; ^3^Department of Emergency, Wuhan Children's Hospital, Tongji Medical College, Huazhong University of Science and Technology, Wuhan, China; ^4^Health Care Department, Wuhan Children's Hospital, Tongji Medical College, Huazhong University of Science and Technology, Wuhan, China; ^5^Department of Radiology, Wuhan Children's Hospital, Tongji Medical College, Huazhong University of Science and Technology, Wuhan, China; ^6^Department of Rehabilitation, Wuhan Children's Hospital, Tongji Medical College, Huazhong University of Science and Technology, Wuhan, China; ^7^Department of Laboratory Medicine, Wuhan Children's Hospital, Tongji Medical College, Huazhong University of Science and Technology, Wuhan, China; ^8^Tianjin Children's Hospital (Children's Hospital of Tianjin University), Tianjin, China; ^9^Tianjin Pediatric Research Institute, Tianjin Children's Hospital (Children's Hospital of Tianjin University), Tianjin, China; ^10^Tianjin Key Laboratory of Birth Defects for Prevention and Treatment, Tianjin, China

**Keywords:** acute necrotizing encephalopathy, pediatric patients, neurological sequelae, magnetic resonance imaging, brain lesion

## Abstract

**Background:** Acute necrotizing encephalopathy of childhood (ANE) is a rare but rapidly progressing encephalopathy. Importantly, the exact pathogenesis and evidence-based treatment is scarce. Thus, we aimed to identify the clinical, imaging, and therapeutic characteristics that associated with prognosis of pediatric ANE patients.

**Methods:** A retrospective study was conducted on pediatric patients with ANE who were admitted to Wuhan Children's Hospital between January 2014 and September 2019. All cases met the diagnostic criteria for ANE proposed by Mizuguchi in 1997. The clinical information and follow-up data were collected. The prognostic factors were analyzed by trend chi-square test and Goodman–Kruskal gamma test.

**Results:** A total of 41 ANE patients ranging in age from 8.9 to 142 months were included in this study. Seven cases (17%) died, and the other 34 survivors had different degrees of neurological sequelae. Factors tested to be significantly correlated with the severity of neurological sequelae were the intervals from prodromal infection to acute encephalopathy (G = −0.553), conscious disturbance (*r* = 0.58), endotracheal intubation (*r* = 0.423), elevation of alanine aminotransferase (*r* = 0.345), aspartate aminotransferase (*r* = 0.393), and cerebrospinal fluid protein (*r* = 0.490). In addition, dynamic magnetic resonance imaging (MRI) evaluation on follow-up revealed that the total numbers of brain lesion location (χ^2^ = 6.29, *P* < 0.05), hemorrhage (*r* = 0.580), cavitation (*r* = 0.410), and atrophy (*r* = 0.602) status were significantly correlated with the severity of neurological sequelae, while early steroid therapy (*r* = −0.127 and 0.212, respectively) and intravenous immunoglobulin (IVIG) (*r* = 0.111 and −0.023, respectively) within 24 h or within 72 h after onset showed no association.

**Conclusions:** Intervals from prodromal infection to acute encephalopathy (≤1 day), total numbers of brain lesion location (≥3), the recovery duration of hemorrhage and atrophy (>3 months), and the presence of cavitation predict severe neurological sequelae in pediatric patients with ANE. Early treatments, including steroid therapy and IVIG, had no correlation with better outcomes. Further studies are needed to establish a consensus guideline for the management of ANE.

## Introduction

Acute necrotizing encephalopathy (ANE) is a rare but acute and fulminant severe encephalopathy, most commonly observed in children with mortality rate up to 30% (1). Less than 10% of patients completely recover, and survivors are often left with severe neurological sequelae (2). Although the exact pathogenesis of ANE remains obscure, it is generally considered to be a parainfectious disease triggered mainly by viral infections, influenza virus and human herpesvirus 6 are two most commonly types (3). Owning to no recommended therapies for ANE until now, symptomatic treatments including antiviral therapy, immunomodulatory agents (like steroids and intravenous immunoglobulin), and therapeutic hypothermia have been used empirically in most cases (4). However, results from different studies were conflicting, and there is still no consensus on these therapies (4–6). Thus, it is important to understand the association of clinical, neuroradiological, and therapeutic characteristics to neurological sequelae of ANE.

A large number of previous studies have investigated the factors that influence the prognosis of ANE; the ANE severity scale (ANE-SS) proposed by Yamamoto et al. (7) in 2015 and the brain Magnetic Resonance Imaging (MRI) scoring system proposed by Wong et al. (8) in 2006 have been accepted widely. However, with the increasing awareness of ANE, more and more pediatric patients are identified and treated promptly/early, whether these factors still meet the clinical needs are unknown. Therefore, we conducted the present study to investigate the association of clinical, radiological, and therapeutic characteristics to prognosis of 41 pediatric ANE patients.

## Materials and Methods

### Study Design and Patients

We performed a retrospective review of the clinical and follow-up data of 41 pediatric patients with ANE admitted to Wuhan Children's Hospital, Tongji Medical College, Huazhong University of Science and Technology, from January 2014 to September 2019. All cases met the diagnostic criteria for ANE proposed by Mizuguchi et al. (9) in 1997, which consist of (1) encephalopathy preceded by viral febrile illness with rapid deterioration in the level of consciousness and convulsions; (2) absent cerebrospinal fluid (CSF) pleocytosis; (3) symmetric multifocal brain lesions; (4) elevation in serum aminotransferase levels; and (5) exclusion of similar diseases. The differential diagnoses were excluded based on clinical and radiological features as well as metabolic screening.

This study was reviewed and approved by the medical ethical committee of Wuhan Children's Hospital, Huazhong University of Science and Technology (2019017). All patients gave written consent (provided by at least a parent or guardian) to the passive use of their medical records for research purposes.

### Clinical Data Collection

The following clinical data were collected: (1) general information such as sex, age, onset age and season, personal history, past history, and family history; (2) clinical features on admission such as etiology of the prodromal infection, intervals from the prodromal infection to acute encephalopathy of ANE, symptoms, etc.; (3) laboratory data on admission such as platelet count (PLT), alanine aminotransferase (ALT), aspartate transaminase (AST), lactate dehydrogenase (LDH), creatine kinase (CK), cerebrospinal fluid (CSF) protein level, and activated partial thromboplastin time (APTT); (4) MRI evaluation; (5) treatment modalities such as methylprednisolone pulse therapy, intravenous immunoglobulin (IVIG), therapeutic hypothermia, plasma exchange, admission to Intensive Care Unit (ICU), and endotracheal intubation; (6) follow-up of all survivors at 2 weeks, 1 month, 3 months, 6 months, and 12 months or longer after the onset of ANE and follow-up evaluation including brain MRI evaluation and motor and cognitive assessment.

### Brain MRI Evaluation

Brain MRI was performed on a 3.0-T scanner. Imaging protocol included axial and sagittal T1-weighted images (T1WI), T2-weighted images (T2WI), diffusion-weighted imaging (DWI), and fluid-attenuation inversion recovery (FLAIR). MRI images of all cases were analyzed independently by two trained neuroradiologists, and a consensus conclusion was finally reached. MRI evaluation includes location of lesions, presence of hemorrhage, cavitation, and atrophy, based on both the initial and follow-up scans. Locations of lesions including thalamus, periventricular, basal ganglia, brainstem, cortex and subcortical of cerebral (frontal, temporal, parietal, and occipital), and cerebellum. Hemorrhage was assessed on T1-weighted images; the presence of hemorrhage was defined as acute/subacute hematoma visualized on the initial study and/or subacute/chronic hematoma on the follow-up study. Cavitation were defined as those having a sharply marginated border, with homogeneous and marked hypointensity on T1-weighted images and CSF-like intensity on T2-weighted images. Atrophy was defined as brain volume decreases that are shown on MRI as cortical sulcus dilation and ventricular dilation.

### Outcome Assessment

Outcome of surviving children was determined 1 year or longer after the onset of ANE based on both cognitive and motor impairments. Cognitive impairment was categorized as normal (DQ/IQ ≥ 70), mild (50 ≤ DQ/IQ < 70), moderate (30 ≤ DQ/IQ < 50), and severe (DQ/IQ < 30) according to the Gesell Developmental Scales (applicable to children <6 years old) or Wechsler Intelligence Test (applicable to children ≥6 years old). Motor impairment was categorized as normal, mild (walking with or without support), moderate (sitting with or without support), and severe (required support to sit and stand). Cognitive and motor assessments were completed separately by the attending pediatric neurologist and rehabilitation physician. The severity of neurological sequelae was categorized by the high degree occurring in either cognitive or motor assessment. For example, neurological outcome was severe in a child with mild motor impairment and severe cognitive impairment.

### Statistical Analysis

The Shapiro–Wilk test and Skewness/Kurtosis test were used to test whether continuous data (such as age onset) meet to be normal distribution, the mean ± SD (such as age onset), interquartile range (i.e., total numbers of lesion location), and frequency (percentage) (i.e., gender, season onset) were used for statistical description for data meets normal, non-normal, or discrete distribution. The one-way ANOVA and Kruskal–Wallis test method were used to determine the differences between groups for grouping factors meeting normal or non-normal distribution, respectively. Coefficient of correlation between ordinal-R^*^C cross-tables was calculated by Goodman–Kruskal gamma or Spearman test. Fisher exact probability test was applied to explore the correlation between brain imaging evolution and neurological sequelae of ANE. All the data management and statistical analysis were performed in SPSS22.0; *P* < 0.05 was considered as statistically significant.

## Results

### Epidemiological, Demographical, and Clinical Characteristics of 41 Pediatric Patients With ANE

Among 41 pediatric patients with ANE, 23 were male (56%) and 18 were female (44%). The patients' ages ranged from 8.9 to 142 months (mean, 52.9 months; median, 40 months; standard deviation, 38.7), and 24 of 41 (59%) patients were under 4 years old. The onset season is more common in winter and spring (56%, from December to May of the following year). There were no family history of similar disease.

As shown in Table 1, the main prodromal symptoms included signs of gastroenteritis (49%) and respiratory infections (46%). Twenty-five cases (61%) were found to have viral infections, with influenza virus (eight cases, 20%) being the most common, and the rest including cytomegalovirus, *Mycoplasma pneumoniae*, Epstein–Barr virus, and human bocavirus. ANE appeared rapidly after the prodromal infection (71% ≤2 days), Thirty-two cases (78%) were in coma, 32 cases (78%) had seizures, and 7 cases (17%) had status epilepticus. With the progression of ANE, 27 (66%) cases had multiorgan dysfunction, 5 (12%) cases had disseminated intravascular coagulation (DIC), and shock occurred in 4 (10%) cases.

**Table 1:** Clinical characteristics of 41 pediatric patients with ANE.

	**Patients**
	**(*n* = 41)**
**Age, median (IQR) (m)**	53.99 (8.9–142)
≤48	24 (59%)
>48	17 (41%)
**Sex**	
Male	23 (56%)
Female	18 (44%)
**Morbidity season**	
Winter–Spring (December–May)	23 (56%)
Summer–Autumn (June–November)	18 (44%)
**Symptoms of prodromal infection**	
Fever	41 (100%)
Respiratory symptoms	19 (46%)
Digestive symptoms	20 (49%)
Other symptoms (rash, fatigue, headache, dizziness)	7 (17%)
**Etiology**	
Influenza virus infection	8 (20%)
Other etiological infections (*Mycoplasma pneumoniae*, Epstein–Barr virus, cytomegalovirus, human bocavirus)	17 (41%)
No clear etiological infection	16 (39%)
**Intervals from prodromal infection to acute encephalopathy (*****d*****)**	
1	13 (32%)
2	16 (39%)
3	8 (20%)
4	3 (7%)
5	1 (2%)
**Presentation of acute encephalopathy**	
Seizures with status epilepticus	7 (17%)
Seizures without status epilepticus	25 (61%)
No seizures	9 (22%)
Lethargy	4 (10%)
Stupor	5 (12%)
Mild coma	19 (46%)
Deep coma	13 (32%)
MODS	27 (66%)
Shock	4 (10%)
DIC	5 (12%)
**Laboratory findings**	
PLT <100 × 10^9^/L	3 (7%)
ALT > 50 U/L	25 (61%)
AST > 50 U/L	32 (78%)
LDH > 300 U/L	31 (76%)
CK > 170 U/L	30 (73%)
APTT > 39 s	17 (41%)
CSF protein > 60 mg/dl	24 (59%)
**Location of lesions**	
Thalamus	41 (100.00%)
Periventricular	34 (83%)
Basal ganglia	26 (63%)
Brainstem	31 (76%)
Cerebral cortex and subcortex	20 (49%)
Cerebellum	18 (44%)
**Radiological findings on follow-Up MRI**^**a**^	
Hemorrhage	28/34 (82%)
Cavitation	28/34 (82%)
Atrophy	21/34 (62%)
**Therapy**	
Methylprednisolone pulse	41 (100%)
IVIG	38 (93%)
Hypothermia	24 (59%)
Plasma exchange	0 (0%)
Admission to ICU	28 (68%)
Endotracheal intubation	9 (22%)
**Prognosis**	
Death	7 (17%)
Survival	34 (83%)
Mild sequelae	8/34 (24%)^a^
Moderate sequelae	12/34 (35%)^a^
Severe sequelae	14/34 (41%)^a^

*m, month; d, day; IQR, interquartile range; MODS, multiple organ dysfunction syndrome; DIC, disseminated intravascular coagulation; ICU, intensive care unit; PLT, Platelet count; ALT, alanine aminotransferase; AST, aspartate transaminase; LDH, lactase dehydrogenase; CK, creatine kinase; APTT, activated partial thromboplastin time; CSF, cerebrospinal fluid; MRI, magnetic resonance imaging; IVIG, intravenous immune globulin*.

a *Data in 34 survivors*.

In laboratory findings, three cases (7%) had decreased platelet counts, 25 cases (61%) had increased ALT, 32 cases (78%) had increased AST, 31 cases (76%) had increased LDH, and 30 cases (73%) had increased CK. APTT was prolonged in 17 cases (41%), and 24 cases (59%) had elevated CSF protein.

Brain MRI study was performed in all cases on admission, 34 survivors had follow-up MRI study at 2 weeks, 1 month, 3 months, and 6 months after the onset; 24 survivors had follow-up MRI study 1 year or longer after the onset. Lesions were frequently observed in thalamic (100%), periventricular (83%), brainstem (76%), basal ganglia (63%), cerebral cortex and subcortex (49%), and cerebellum (44%). The total cumulative number of lesion locations was 4 with an IQR of 3–5.5.

All pediatric patients (100%) were treated with methylprednisolone pulse therapy (20 mg/kg daily for 5 days, then with oral prednisolone taper over 1 month); steroids were administered within 24 h after onset in 8 cases (19.5%) and within 72 h in 22 cases (54%). Thirty-seven cases (90%) received IVIG (1 g/kg daily for 2 days), 24 cases (59%) with therapeutic hypothermia, and none received plasma exchange. Twenty-eight (68%) cases were admitted to ICU, and nine (22%) cases received endotracheal intubation.

Among the 41 pediatric ANE patients, 7 patients (17%) died, and the remaining 34 survivors had different degrees of neurological sequelae, predominantly moderate and severe types (mild, 24%; moderate, 35%; severe, 41%).

### The Association Between Clinical Characteristics and ANE Survival Status

In seven patients who died (male/female = 5:2), six had coinfections, six had prodromal infection and the duration to acute onset was within 2 days (three of seven ≤ 1 day), six had status epilepticus, and six had multiorgan failure. The distribution of clinical features, laboratory findings, location of lesions on brain MRI, and treatment modalities between dead and surviving patients showed no significant differences.

### The Association Between Clinical Characteristics and Neurological Sequelae of ANE

**Figure 1:**
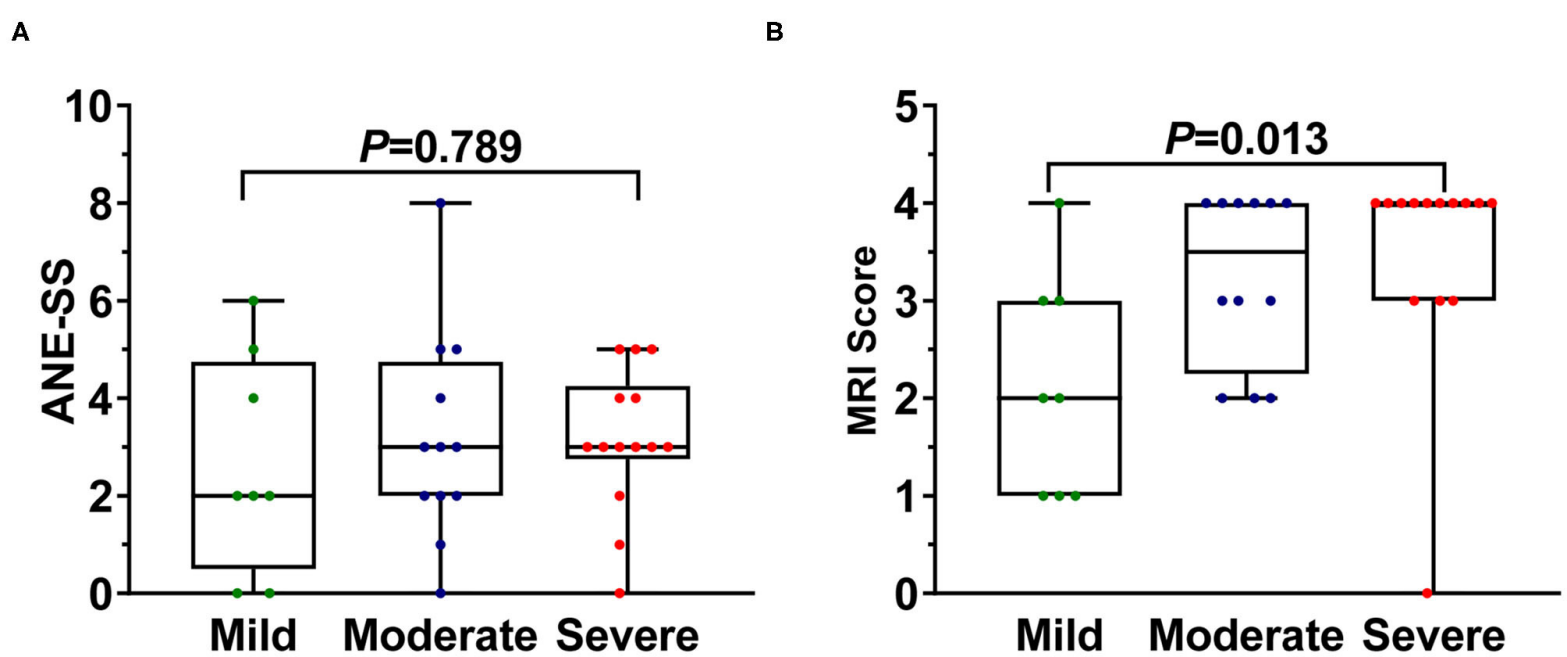
The difference of **(A)** ANE-SS and **(B)** MRI scoring system in 34 survival ANE patients with varying degrees of neurological sequelae. The one-way ANOVA (ANE-SS) and Kruskal–Wallis test (MRI scoring system) method were used to determine the differences among groups.

The association of ANE-SS score (7) to the neurological sequelae of 34 survivors were first evaluated and showed no significant distribution difference (*P* = 0.789, Figure 1A). Thus, the clinical features and laboratory findings were then systematically analyzed in 34 survivors (Tables 2, 3). The intervals from the prodromal infection to acute encephalopathy and the degree of conscious disturbance were associated with the severity of neurological sequelae, and the coefficient G values were −0.553 and 0.580, respectively (*P* < 0.05). Importantly, the intervals from the prodromal infection to acute encephalopathy was <3 days in 92% patients with moderate and 100% patients with severe neurological sequelae. Trend chi-square test revealed that endotracheal intubation was significantly correlated with the severity of neurological sequelae (*r* = 0.423, *P* < 0.05). In addition, the increase in ALT, AST, and CSF protein were also significantly associated with the degree of neurological sequelae in ANE (*r* = 0.345, 0.393, and 0.490, respectively, *P* < 0.05).

**Table 2:** The association between clinical manifestations and neurological sequelae of ANE.

**Clinical manifestations**		**Neurological sequelae**			***G/r***	***P-*value**
		**Mild (*n* = 8)**	**Moderate (*n* = 12)**	**Severe (*n* = 14)**		
Intervals from prodromal infection to acute encephalopathy (number, %)	≤1 day	2 (25%)	1 (8.3%)	6 (42.9%)	−0.553^a^	*0.035*
	2–3 days	3 (37.5%)	10 (83.3%)	8 (57.1%)		
	4–5 days	3 (37.5%)	1 (8.3%)	0 (0)		
Consciousness disturbance	Somnolence	2 (25%)	1 (8.3%)	0 (0)	0.580^a^	*0.002*
	Stupor	2 (25%)	3 (25%)	0 (0)		
	Mild coma	3 (37.5%)	5 (41.7%)	8 (57.1%)		
	Deep coma	1 (12.5%)	3 (25.0%)	6 (42.9%)		
Tracheal intubation	No	8 (100%)	11 (91.7%)	8 (57.1%)	0.423^b^	*0.024*
	Yes	0 (0)	1 (8.3%)	6 (42.9%)		

a *Use Goodman–Kruskal gamma method to test*.

b *Use Spearman correlation test*.

**Table 3:** The association between laboratory findings and neurological sequelae of ANE.

**Laboratory findings**		**Neurological sequelae**			***r***	***P-*value**
		**Mild (*n* = 8)**	**Moderate (*n* = 12)**	**Severe (*n* = 14)**		
ALT	Normal	5 (62.5%)	6 (50%)	3 (21.4%)	0.345^a^	*0.045*
	High	3 (37.5%)	6 (50%)	11 (78.6%)		
AST	Normal	4 (50%)	4 (33.3%)	1 (7.1%)	0.393^a^	*0.022*
	High	4 (50%)	8 (66.7%)	13 (92.9%)		
CSF protein	Normal	5 (62.5%)	6 (50%)	1 (7.1%)	0.490^a^	*0.003*
	High	3 (37.5%)	6 (50%)	13 (92.9%)		

*ALT, alanine aminotransferase; AST, aspartate transaminase; CSF, cerebrospinal fluid*.

a *Use Spearman correlation test*.

### The Association of Brain Lesions on MRI to Neurological Sequelae of ANE

**Figure 2:**
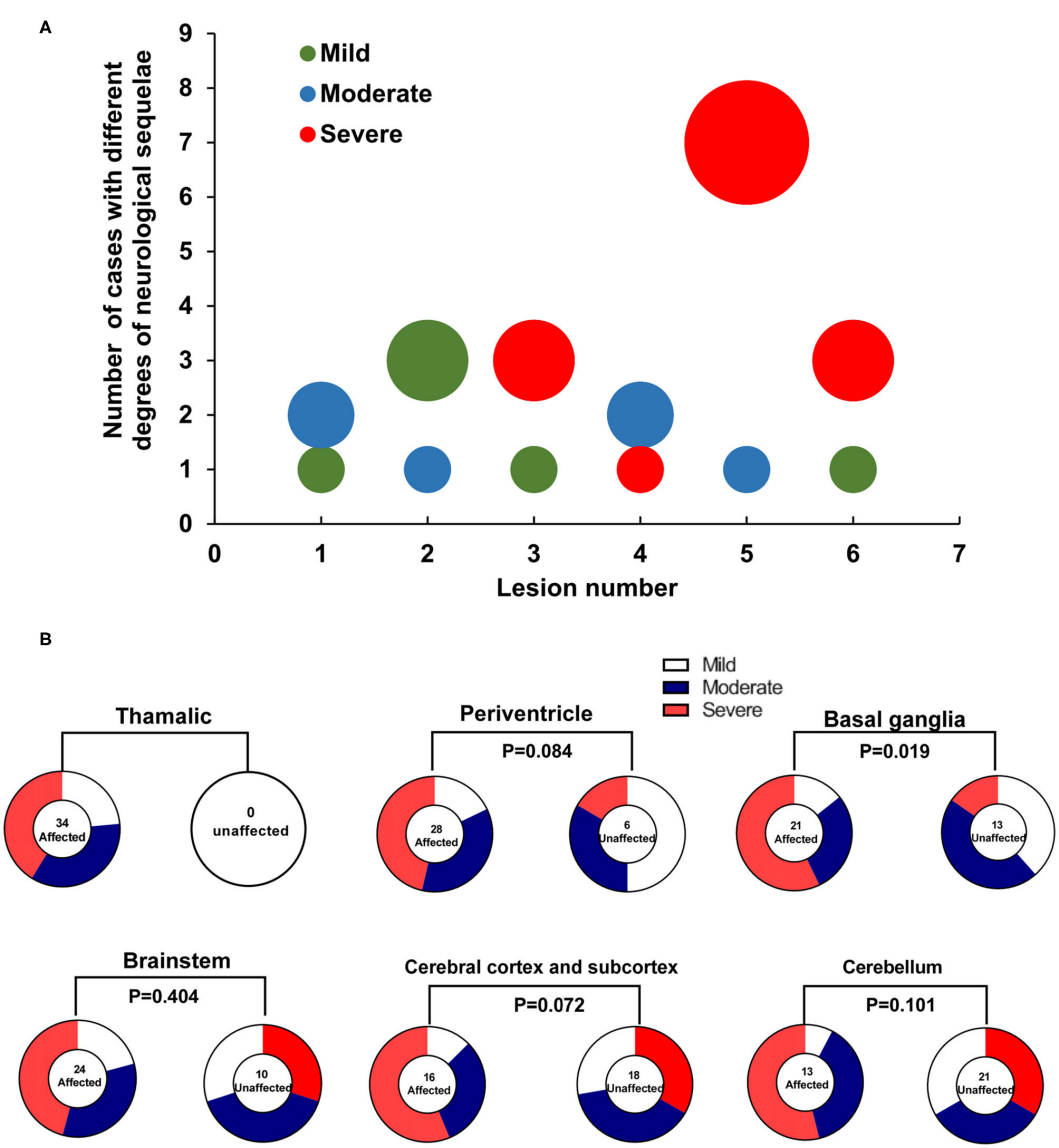
The association of lesions in brain to the severity of neurological sequelae in ANE patients. **(A)** Distribution difference in the number of brain lesion locations in 34 survival ANE patients with varying degrees of neurological sequelae. **(B)** The association of brain lesion locations to the severity of neurological sequelae in 34 survival ANE patients.

ANE patients with severe neurological sequelae were found to have significantly higher MRI scores than those with moderate and mild neurological sequelae [quartile distribution: severe, 4 (3, 4); moderate, 3.5 (2.3, 4); mild, 2 (1, 3) (χ^2^ = 8.74, *P* = 0.013, Figure 1B) *via* using the MRI scoring system proposed by Wong et al. (8), while no significant difference was shown between moderate and mild groups. Interestingly, the number of lesion locations in the brain with severe outcome was significantly higher than that of children with moderate and mild outcomes. Quartile distribution were severe 5 (3.8, 5.3) > moderate 3.5 (2.3, 5.8) > mild 2.5 (2, 4), with significant difference (χ^2^ = 6.29, *P* < 0.05, Figure 2A). The brain lesion in 100% patients with severe neurological sequelae occurred in more than two sites (3–6 numbers). Furthermore, lesion in basal ganglia (*r* = 0.9, *P* = 0.019) was positively associated with the severity of neurological sequelae, while lesions in the white matter (including cerebral cortex and subcortex, periventricular, and cerebellum) and brainstem that were important for MRI scoring system showed no obvious association (Figure 2B).

### The Association of Neuroimaging Evolution and Neurological Sequelae of ANE

**Figure 3:**
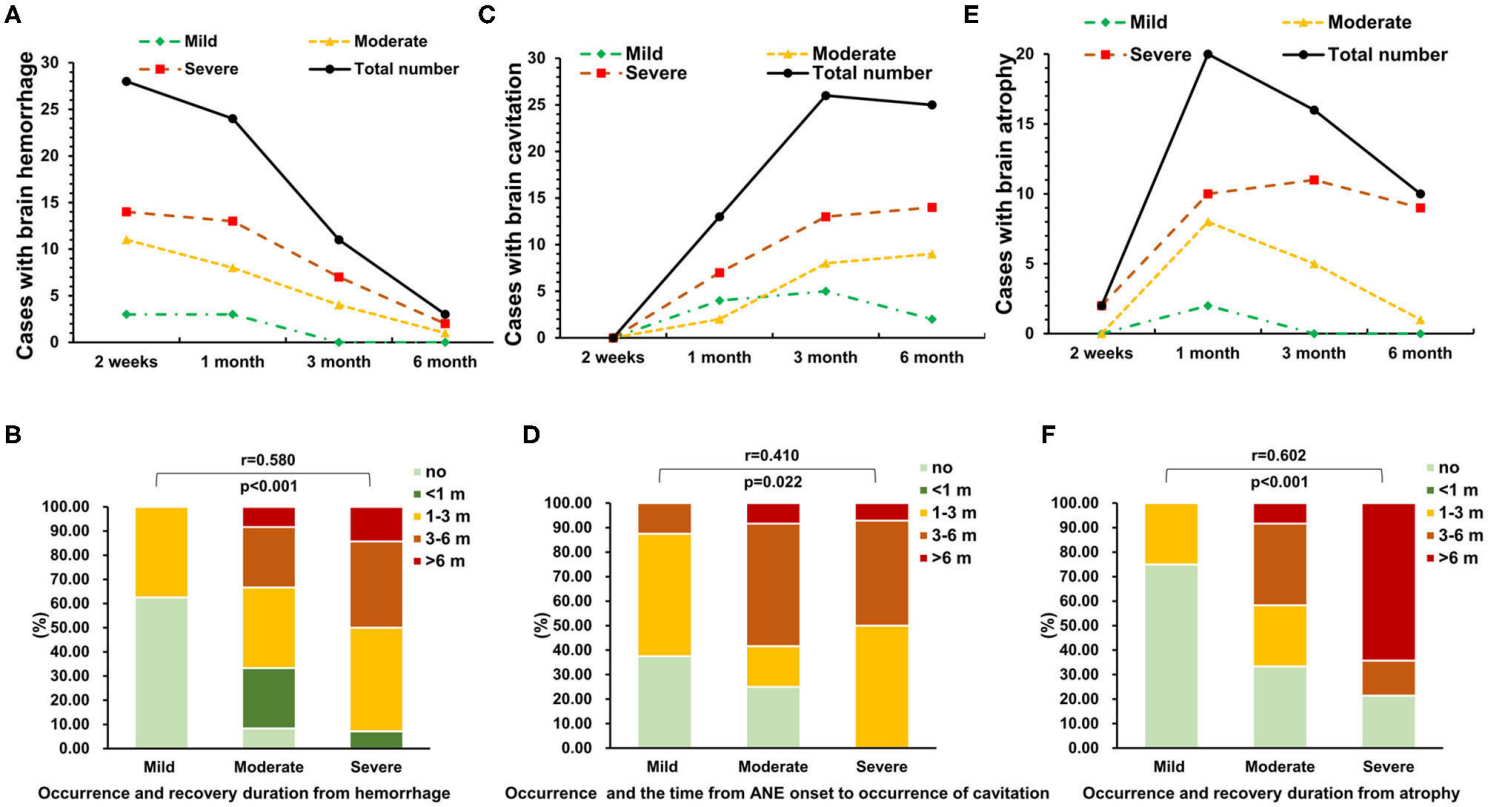
The association of neuroimaging evolution and neurological sequelae of ANE. The dynamic changes in **(A,B)** brain hemorrhage, **(C,D)** cavitation, and **(E,F)** atrophy in 34 survival ANE patients with different degrees of neurological sequelae during the follow-up.

The majority of survivors (28/34, 82%) with ANE had hemorrhage in brain MRI at 2 weeks, which gradually disappeared within 1–3 months, and most of them showed no hemorrhage by 6 months (Figure 3A). The occurrence rate and recovery duration from hemorrhage in survivors with moderate and severe neurological sequelae were significantly higher than that in mild cases (*r* = 0.580, *P* < 0.001, Figure 3B). Recovery duration of hemorrhage longer than 3 months occurred in moderate and severe cases. Cavitation occurred in 28 of 34 (82%) survivors during the full follow-up, reaching in peak after 3 months (Figure 3C). The occurrence rate and the time from ANE onset to occurrence of cavitation were significantly higher in severe cases than that in mild and moderate cases (*r* = 0.410, *P* = 0.022, Figure 3D). Brain atrophy appeared in 21 of 34 (62%) survivors after 1 month (Figure 3E). The occurrence rate and recovery duration of brain atrophy in moderate and severe cases were significantly higher than that in mild cases (*r* = 0.602, *P* < 0.001, Figure 3F). In addition, the recovery duration of brain atrophy longer than 3 months occurred in moderate and severe cases.

**Figure 4:**
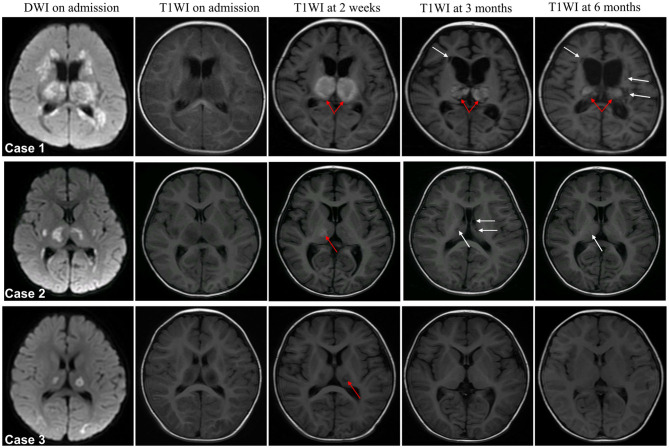
Representative dynamic MRI images of survival ANE patients with different degrees of neurological sequelae. Case 1 with severe, case 2 with moderate and case 3 with mild neurological sequelae. Arrow in red indicates hemorrhage and white indicates cavitation.

In patients with severe neurological sequelae, hemorrhage almost spanned the full follow-up period (Figure 4, case 1 occurred in 2 weeks after admission and persist during the course of follow-up), while it disappeared in middle stage of patients with moderate neurological sequelae (Figure 4, case 2); it was only observed in the subacute stage in the mild case (Figure 4, case 3). Atrophy was across the full follow-up in patients with severe neurological sequelae (case 1), and no one had atrophy in moderate and mild cases (cases 2 and 3). In patients with moderate and severe neurological sequelae, cavitation occurred in the middle stage and persistently existed in severe cases (cases 1 and 2) and almost disappeared in the late stage of moderate cases (case 2). Importantly, no one had cavitation in the mild case during the full disease course (case 3).

### The Association of Activation Time of Steroid and IVIG Therapies to Neurological Sequelae of ANE

Most of the cases received early empirical interventions (within 72 h after onset). The mean interval from onset to steroid therapy activation was 4 (2, 5) days, 20% (eight of 41 cases) started steroid therapy within 24 h after onset, and 54% (22 of 41 cases) started steroid therapy within 72 h after onset. Of the 34 survivors, 15% (five of 34 cases) started steroid therapy within 24 h after onset, and 47% (16 of 34 cases) started steroid therapy within 72 h after onset. In addition, 47% (16 of 34 cases) started IVIG within 24 h after onset, and 76% (26 of 34 cases) started IVIG within 72 h after onset. However, early steroid therapy and IVIG (within 24 or 72 h after onset) showed no significant association to the neurological sequelae of ANE patients (Table 4).

**Table 4:** The association between steroid and IVIG therapy activation time and neurological sequelae of ANE.

**Therapy**	**Time**	**Neurological Sequelae**			**R**	***P*-value**
		**Mild (*n* = 8)**	**Moderate (*n* = 12)**	**Severe (*n* = 14)**		
Steroid	≤24 h	1 (12.5%)	1 (8.3%)	3 (21.4%)	−0.127^a^	*0.176*
	>24 h	7 (87.5%)	11 (91.7%)	11 (78.6%)		
	≤72 h	5 (62.5%)	6 (50%)	5 (35.7%)	0.212^a^	*0.166*
	>72 h	3 (37.5%)	6 (50%)	9 (64.3%)		
IVIG	No	1 (12.5%)	1 (8.3%)	1 (7.1%)	0.111^a^	*0.171*
	≤24 h	4 (50%)	6 (50%)	6 (42.9%)		
	>24 h	3 (37.5%)	5 (41.7%)	7 (50%)		
	No	1 (12.5%)	1 (8.3%)	1 (7.1%)	−0.023^a^	*0.190*
	≤72 h	5 (62.5%)	10 (83.3%)	11 (78.6%)		
	>72 h	2 (25%)	1 (8.3%)	2 (14.3%)		

a *Use Spearman correlation test*.

## Discussion

ANE is an acute, fulminant, severe encephalopathy first reported by Mizuguchi et al. (10) in 1995, with rapid disease progression, devastation, high mortality, and high disability. Previous literature has reported a mortality rate of up to 30%, with 15–35% survivors often have severe degree of neurological sequelae (2, 11–13). In the present study, the mortality rate of ANE (17%) was lower than that of previous reports (2, 13). Administration of steroids at the early stage of ANE (within 24 or 72 h after onset) has been reported to be associated with a better prognosis (1, 4), However, early intervention (including steroid and IVIG) showed no significant association to the severity of neurological sequelae, which may explain the result that the proportion of severe neurological sequelae in this study (41%) was similar to previous studies. Thus, the increased recognition and early empirical intervention, steroid, and IVIG may improve the survival outcome of ANE but not the neurological sequelae.

The ANE-SS (7) considered that age over 48 months had a poor prognosis, while other studies (3, 9) reported that children over 4 years tended to have a better prognosis and children under 1 year had a poorer prognosis. In this study, neither age nor sex were associated with neurological sequelae of ANE, which further supported the conclusion that there was no significant correlation between age and the prognosis of ANE obtained by Lee et al. (11).

Influenza virus has been considered as the most common cause for the initiation of ANE (14), while Okumura et al. (15) found no difference in clinical features and outcome in 11 ANE cases secondary to influenza as compared with 11 non-influenza cases. In the present study, 60% of the cases were found to have prodromal infection, of which influenza virus accounted for 20%. However, there was no significant association between each etiological infection and prognosis, same as the findings obtained by Okumura et al. (15) and Lee et al. (11). In this regard, development of ANE seems to be independent of the type of infectious agents. Instead, our study found that the intervals from prodromal infection to acute encephalopathy was significantly correlated with the degrees of neurological sequelae (≤1 day), indicating that viral infection may correlate the neuron recovery in ANE and with the potential to be used as indicator to predict disease recovery.

A number of studies have shown that some laboratory parameters associated with the prognosis of ANE, such as the serum ALT and protein in CSF (16, 17). Specifically, platelet count <100,000/μl (score of 1) and shock (score of 3) are important factors indicating poor prognosis in ANE-SS (7). In this study, elevated ALT, AST, and CSF protein, but not decreased platelet count and shock, were found to be associated with the severity of neurological sequelae in ANE. The suspected reason may be that all of the cases in this study were treated with hormones and IVIG in the early stages, and thus, shock and DIC only occurred in rare cases.

Brain MRI is an important index for evaluating the prognosis of ANE, and several studies (8, 11) have identified that brainstem lesions are an important factor for the poor prognosis of ANE. Brainstem lesions can be followed by severe brainstem dysfunction characterized by respiratory insufficiency, cardiopulmonary failure, and neurogenic pulmonary edema, which may explain the high mortality rate of the disease. Wong et al. (8) created an MRI scoring system that included location of lesions in the brainstem and the white matter (including cerebral and cerebellar), hemorrhage, and cavitation, with each score of 1. However, this MRI scoring system showed no difference between moderate and mild cases in this study, while the total number of lesion locations was significantly higher in ANE patients with severe neurological sequelae. Furthermore, lesions in the basal ganglia, but not the brainstem and the white matter, was associated with the severity of neurological sequelae, which indicated that the more brain locations are involved, the poorer is the prognosis.

Hemorrhage, atrophy, and cavitation are destructive processes to brain tissues (2). In this study, the occurrence rate, recovery duration from hemorrhage and atrophy (≥3 months), and the time from ANE onset to occurrence of cavitation were associated with severe neurological sequelae. Thus, although the MRI scoring system for Wong et al. (8) is feasible, the dynamic changes in hemorrhage, atrophy, and cavitation observed by MRI may be more valuable for the prediction of neurological sequelae severity in pediatric patients with ANE.

In conclusion, this study provides us with a better understanding of factors associated with the prognosis of pediatric patients with ANE, including survival status and neurological sequelae. Its poor prognostic factors are mainly related to the number of lesion locations in the brain (≥3), especially lesions in the basal ganglia, intervals from prodromal infection to acute encephalopathy (≤1 day), the occurrence and recovery duration of hemorrhage and atrophy (>3 months), and the presence of cavitation. The widely used empirical interventions (including steroid and IVIG) at the early stage of ANE have no correlation with better outcomes. This study also has some limitations. First, it was a retrospective study with inconsistent follow-up time points, and thus, the causal relationship between the tested parameters and the prognosis of ANE could not be confirmed. Last and most importantly, most of these patients did not have genetic analysis; future studies should address whether genotype influences outcomes or not.

## Data Availability Statement

The raw data supporting the conclusions of this article will be made available by the authors, without undue reservation.

## Ethics Statement

The studies involving human participants were reviewed and approved by Medical Ethics Committee of Wuhan Children's Hospital, Tongji Medical College, Huazhong University of Science & Technology (2019017). Written informed consent to participate in this study was provided by the participants' legal guardian/next of kin. Written informed consent was obtained from the minor(s)' legal guardian/next of kin for the publication of any potentially identifiable images or data included in this article.

## Author Contributions

C-QC, Z-SL, J-SH, and G-FW: study conception and design. H-MZ, S-MZ, M-QL, H-JM, and TL: data acquisition. CY and H-MZ: analysis and data interpretation. H-MZ and C-HY: drafting of the manuscript. All authors contributed to the article and approved the submitted version.

## Conflict of Interest

The authors declare that the research was conducted in the absence of any commercial or financial relationships that could be construed as a potential conflict of interest.

## References

[1] Bassuk AG Burrowes DM McRae W. Acute necrotizing encephalopathy of childhood with radiographic progression over 10 hours. Neurology. (2003) 60:1552–3. 10.1212/01.WNL.0000058757.52327.17 12743257

[2] Zhu HM Liu ZS. Advances in clinical and imaging studies of acute necrotizing encephalopathy. Zhonghua er ke za zhi. (2017) 55:865–8. 10.3760/cma.j.issn.0578-1310.2017.11.017 29141322

[3] Wu X Wu W Pan W Wu L Liu K Zhang HL. Acute necrotizing encephalopathy: an underrecognized clinicoradiologic disorder. Mediat Inflam. (2015) 2015:792578. 10.1155/2015/792578 25873770 PMC4385702

[4] Bashiri FA Al Johani S Hamad MH Kentab AY Alwadei AH Hundallah K. Acute necrotizing encephalopathy of childhood: a multicenter experience in Saudi Arabia. Front Pediatr. (2020) 8:526. 10.3389/fped.2020.00526 33163461 PMC7581867

[5] Okumura A Mizuguchi M Kidokoro H Tanaka M Abe S Hosoya M. Outcome of acute necrotizing encephalopathy in relation to treatment with corticosteroids and gammaglobulin. Brain Dev. (2009) 31:221–7. 10.1016/j.braindev.2008.03.005 18456443

[6] Seo HE Hwang SK Choe BH Cho MH Park SP Kwon S. Clinical spectrum and prognostic factors of acute necrotizing encephalopathy in children. J Korean Med Sci. (2010) 25:449–53. 10.3346/jkms.2010.25.3.449 20191046 PMC2826728

[7] Yamamoto H Okumura A Natsume J Kojima S Mizuguchi M. A severity score for acute necrotizing encephalopathy. Brain Dev. (2015) 37:322–7. 10.1016/j.braindev.2014.05.007 24931733

[8] Wong AM Simon EM Zimmerman RA Wang HS Toh CH Ng SH. Acute necrotizing encephalopathy of childhood: correlation of MR findings and clinical outcome. Am J Neuroradiol. (2006) 27:1919–23. 17032866 PMC7977901

[9] Mizuguchi M. Acute necrotizing encephalopathy of childhood: a novel form of acute encephalopathy prevalent in Japan and Taiwan. Brain Dev. (1997) 19:81–92. 10.1016/S0387-7604(96)00063-0 9105653

[10] Mizuguchi M Abe J Mikkaichi K Noma S Yoshida K Yamanaka T. Acute necrotising encephalopathy of childhood: a new syndrome presenting with multifocal, symmetric brain lesions. J Neurol Neurosurg Psychiatry. (1995) 58:555–61. 10.1136/jnnp.58.5.555 7745402 PMC1073485

[11] Lee CG Kim JH Lee M Lee J. Clinical outcome of acute necrotizing encephalopathy in related to involving the brain stem of single institution in Korea. Korean J Pediatr. (2014) 57:264–70. 10.3345/kjp.2014.57.6.264 25076971 PMC4115067

[12] Kim JH Kim IO Lim MK Park MS Choi CG Kim HW. Acute necrotizing encephalopathy in Korean infants and children: imaging findings and diverse clinical outcome. Korean J Radiol. (2004) 5:171–7. 10.3348/kjr.2004.5.3.171 15467414 PMC2698159

[13] Marco EJ Anderson JE Neilson DE Strober JB. Acute necrotizing encephalopathy in three brothers. Pediatrics. (2010) 125:e693–8. 10.1542/peds.2009-1984 20142283 PMC3207236

[14] Nakata K Kashiwagi M Masuda M Shigehara S Oba C Murata S. A child with acute encephalopathy associated with quadruple viral infection. Front Pediatr. (2015) 3:26. 10.3389/fped.2015.00026 25883930 PMC4382965

[15] Okumura A Abe S Kidokoro H Mizuguchi M. Acute necrotizing encephalopathy: a comparison between influenza and non-influenza cases. Microbiol Immunol. (2009) 53:277–80. 10.1111/j.1348-0421.2009.00124.x 19457168

[16] San Millan B Teijeira S Penin C Garcia JL Navarro C. Acute necrotizing encephalopathy of childhood: report of a Spanish case. Pediatric Neurol. (2007) 37:438–41. 10.1016/j.pediatrneurol.2007.07.007 18021928

[17] Wolf K Schmitt-Mechelke T Kollias S Curt A. Acute necrotizing encephalopathy (ANE1): rare autosomal-dominant disorder presenting as acute transverse myelitis. J Neurol. (2013) 260:1545–53. 10.1007/s00415-012-6825-7 23329376

